# Analytical comparison study of the clinical and radiological outcome of spine fixation using posterolateral, posterior lumber interbody and transforaminal lumber interbody spinal fixation techniques to treat lumber spine degenerative disc disease

**DOI:** 10.1186/s13013-015-0040-0

**Published:** 2015-05-27

**Authors:** Moh’d M Al Barbarawi, Ziad M Audat, Mohammed Z Allouh

**Affiliations:** Department of Neuroscience/ Division of Neurosurgery, Level 7A. King Abdullah University Hospital, Jordan University of Science and Technology, Irbid-Amman Street, P.O.box 3030, Irbid, Jordan; Department of orthopeadic, Level 8A. King Abdullah University Hospital, Jordan University of Science and Technology, Irbid-Amman Street, P.O.box 3030, Irbid, Jordan; Department of Anatomy, Faculty of medicine, Jordan University of Science and Technology, Irbid-Amman Street, P.O.box 3030, Irbid, Jordan

**Keywords:** Degenerative disc disease (DDD), Spinal fixation, Transforaminal lumber interbody fusion (TILF), Posterolateral fusion (PLF), Posterior lumber interbody fusion (PLIF)

## Abstract

**Background:**

Degenerative disc disease is a common cause of chronic and disabling back pain that requires surgical intervention, posterolateral and posterior instrumental fixation (PLF), posterior lumber interbody fusion (PLIF) and transforaminal lumber interbody fusion (TLIF) are the techniques used to deal with such a problem.

**Objective:**

To compare the clinical and radiological outcome of the variable surgical techniques used to deal with Lumber degenerative disc disease and to recommend the technique of choice.

**Methods:**

120 patients were treated between 2003 and 2010 at king Abdullah university hospital for lumber disc disease. The patients were divided into three groups: Group I (PLF n = 30 [59 levels]); Group II (PLIF n = 40 [70 levels]); and Group III (TLIF n = 50 [96 levels]). All patients had the same pre- and postoperative clinical and radiological evaluations (using Stanford score and local criteria and Oswestry Disability Index [ODI],). All cases had three months and then yearly for five years follow ups.

**Results:**

There was no observed difference in the rates of intra-operative complications (Group I: 10 %; Group II: 8 %; Group III: 14 %; *p* = 0.566) and postoperative complications (Group I: 13.3 %, Group II:17.5 %, Group III: 18 % with *p* = 0.332). Among the groups. There was a vital decrease in the ODI scores over time (p < 0.005) but no major difference among the groups at different follow-up times. Radiographic fusion rates for Groups I, II and III were 90 %, 92.5 % and 94 %, respectively.

**Conclusions:**

The surgical outcome of PLF, PLIF and TLIF used to treat degenerative disc disease is almost similar, there is no significant differences observed in complications and clinical outcomes. However, TILF may have better radiological outcome.

## Introduction

Degenerative Lumber spine disc disease is a common cause of disabling pain encountered by spine surgeon and requires intervention. A wide variety of symptoms and signs are frequently seen including chronic Low back pain, sciatic pain, paraesthesia, weakness, intermittent claudication and sphencteric disturbances. Several surgical approaches with and without instrumental fixation have been suggested to deal with this entity; posterior lumber interbody fusion (PLIF), transforaminal lumbar interbody fusion (TLIF), and posterolateral fusion and posterior instrumentation (PLF) are commonly utilized [1–4].

The introduction of pedicle screw fixation has provided a direct spinal stability and improved the fusion rate [5, 6]. PILF was first described by Cloward in 1940 and then modified by Lin; it then became widely used [7, 8]. This technique provides a three-column fixation stability with anterior support and 360° fusion, and is approached only from the posterior [9–11]. It also prevents the posterior instruments from strain and failure, and may result in a significant spondylolisthetic reduction [12–14], the posterior approach has lower co- morbidity and cost when compared to the anterior approach [15]. The pitfall of PLIF is the limitation of fusions to L3–S1 to evade the risk of damage to the conus medullaris and cauda equina from traction [16].

Harms et al. recommended TLIF technique to deal with DDD and other spinal pathologies with less rate of complications. It provides more rooms for bone graft application and preserves the posterior tension band, which offers more stability. TILF also augments segmental lordosis when compared to PLIF and makes revision surgery easier because the contralateral foramen is not disturbed [17–20]. In this communication the authors compare the clinical and radiological outcomes of PLF, PLIF and TLIF, the common surgical techniques used to treat degenerative disc disease with lumber canal stenosis and to recommend the technique of choice.

### Statistical package

The Statistical Package for the Social Sciences version 15 (SPSS, Chicago, IL, USA) was used for data processing and analysis. The subjects’ variables were described using frequency distribution for categorical variables, and mean and standard deviation for continuous variables. Chi-square test was used to compare the percentages, and one-way ANOVA was used to compare the means among the three groups. The changes in scores over time were tested using repeated measures analysis. A p-value < 0.05 was considered to be statistically significant.

### Clinical material and study design

The study was approved by ethical committee for human research (IRB) at Jordan University of Science and Technology. The study group consisted of 120 patients (225 levels) treated for lumber spine degenerative disc disease at King Abdullah University Hospital between 2003 and 2010. All cases were treated by same surgeon using the techniques of PLF, PLIF and TILF. Inclusionary criteria included; patients suffered symptomatic lumber pine degenerative disc disease with canal stenosis that failed to respond to all conservative treatment modalities. Exclusionary criteria included: patients with spinal fractures, spondylosteoamyelitis, spondylolisthesis, previous back surgery with instrumentation and cases done by other surgeons.

Different types of instrumental devices were used including polyaxial screws and polyether-etherketone interbody cage. Clinical, radiological and magnetic resonance imaging assessment were the pre operative methods of evaluation to all patients. While all cases were done by a single surgeon; the pre and post operative evaluation was carried out by a different and independent surgeon. The cases were divided into three main groups:

**Group I**: included of 30 patients (with 59 levels) aged 36–69 (mean age 56.2) years who were treated with PLF. The male to female ratio was 12:18. Patients had variable complaints: back pain, sciatica and neurogenic claudication. Sensory disturbance was present in all patients except in two cases, and motor weakness was observed in ten patients. Two patients had urinary retention. The duration period of symptoms ranged 2–25 (mean 7.8) years (Table 1).

**Group II**: consists of 40 patients (with 70 levels) aged 29–73 (mean 51.6) years. The male to female ratio in this group was 12:28. PLIF technique was used in this group. All patients had back pain, sciatica and neurogenic claudication. Sensory disturbance was found in 18 patients, weakness was noted in 15 patients and only three patients had urinary symptoms. The duration period of symptoms ranged from three months to 29 years (mean 6.78 years) (Table 1).

**Group III**: consists of 50 patients (with 90 levels) aged 29–75 (mean 44.9) years who were treated with TLIF technique. The male to female ratio was 19:31. Symptoms included back pain, sciatica, neurogenic intermittent claudication. 31 patients had sensory disturbances, 21 had muscle weakness and one had urinary symptoms. The duration of symptoms extended from 5 months to 20 years (mean 5.4 years) (Table 1).

**Table 1:** Demographic and clinical characteristics of patients according to method of surgery

	PLF	PLIF	TLIF	P-value
	Group 1	Group 2	Group 3	
	(*n* = 30) 59 levels	(*n* = 40) 70 levels	(*n* = 50) 90 levels	
Gender				0.305
Male	12(40)	12 (30)	19 (38)	
Female	18 (60)	28 (70)	31 (62.)	
Age, mean (SD); range	36-69	29-73	29-75	0.072
	(36–69)	51.6	45.9	
Duration of symptoms (Months)	2 m -25y (7.8)	3 m-29y (6.78)	5 m- 20 y (5.4 y)	
Chronic back Pain	24 (80)	35 (87.5)	35 (70)	0.188
Radiating pain				0.006
unilateral	18 (60)	10 (25)	30 (60)	
bilateral	9(30)	20 (50)	15 (30)	
Sensory disturbance				0.608
yes	28 (93.4)	32 (80.)	31 (62)	
bilateral	18	17	7	
unilateral	10	15	24	
no	2 (6.6)	8(20)	19 (38)	
Muscle weakness				0.930
yes	10(33.4)	15(37.5)	21(42)	
no	20 (66.6)	25 (62.5)	29 (68)	
Claudication				0.616
yes	22 (73.3)	30 (75)	45 (90)	
No	8 (26,7)	10 (25)	5 (21.6)	
Sphincter disturbance				0.361
normal	28 (93.3)	37(92.5)	49 (95)	
abnormal	2 (6.7)	3 (7.5)	1 (5)	

Table 1: shows the pre-operative demographic and clinical appearances of patients according to the surgical techniques

All patients were evaluated clinically before surgery and post operatively at interval of three months, one year, two years three years and five years.

The Oswestry Disability Index (ODI), Stanford score [21, 22] besides our provisional clinical criteria were used for outcome assessment. The Local clinical criteria included; subjective pain improvement, patient’s satisfaction from surgery, ability to do shopping alone, practicing exercises and walking unaided, the outcome was classified into four main categories:

**Poor outcome:** was considered when patients reported no change or getting worse postoperatively, pain did not respond to analgesia, increased numbness, paraesthesia, same or increased weakness with dissatisfaction and inability to do daily activities.

**Fair outcome:** was considered when a patient reported some improvement in back pain or sciatica up to 50 % with some analgesia, or mild improvement in numbness, paraesthesia or weakness. However, those patients still had mild difficulty with daily activities but the satisfaction level is increased up to 50 %–70 % as compared with the preoperative status.

**Good outcome:** described when the patient experienced significant improvement in pain (70 %) that required occasional analgesia, a significant improvement in sensory symptoms, remarkable improvement in weakness with no limitation in daily activities. In such cases, patient satisfaction was observed at 70 %–80 %.

**Excellent outcome**: was measured when the patient had no pain, no neurological deficits and no limitation in daily activities, with more than 80 % improvement.

Radiological evaluation included plain X-rays at three months, one year, two, three and five after surgery with dynamic lateral views at the last follow-up. Radiographic fusion was considered to be present based on obliteration of the disc space with continuous bony mass between the two vertebral bodies (PLIF and TLIF), continuous trabecular bone throughout the inter-transverse fusion mass, no motion on flexion and extension radiographs, and absence of instrument loosening or failure (all groups).

### Statistical analysis and results

Table 1 shows the pre-operative demographic and clinical appearances of patients according to the surgical techniques. Preoperatively, the three groups fluctuated significantly (*p* = 0.006) in terms of pain radiation. Patients with unilateral or radiating pain were basically treated with TLIF compared to patients treated with PLF and PLIF. There were no major differences among the three treatment groups in terms of gender (*p* = 0.305), average age (*p* = 0.072), back pain (*p* = 0.188), sensory disturbance (*p* = 0.608), muscle weakness (*p* = 0.930), claudicating pain 0.616 and sphincter disturbance (*p* = 0.361).

The peri operative co- morbidities among the patients according to surgical technique are shown in (Table 2). It appears that the intra-operative rate of complications is insignificant among the three groups (Group I: 10 %; Group II: 8 %; Group III: 14 %; *p* = 0.566). This included a dural tear, blood loss, nerve root injury and minor vascular injury. Intraoperative complications of Group I were mainly small dural tears (less than 1 cm) that appeared during laminectomy and nerve root decompression. In Group II, a small dural tear was noticed in four patients and pedicle fracture in three patient who had some osteoporosis. A nerve root injury ended with foot drop was observed in the third group.

**Table 2:** Intra and post operative complications in all groups

	PLF	PLIF	TLIF	P-value
	Group 1	Group 2	Group 3	
	(n = 30)	(n = 40)	(n = 50)	
Intra operative complications				0.566
No	27 (90)	35 (92)	30 (86)	
Yes	3 (10)	5 (8)	7(14)	
Post operative complications				0.332
No	32 (86.7)	33 (82.5)	41 (82)	
Yes	4 (13.3)	7 (17.5)	9 (18)	
Infection	2	2	3	
Sciatcia	2	3	2	
DVT & PE	0	2	2	
Muscle Weakness	0	0	2	
Mortality rate	0 (0 %)	1(2 %)	1(2 %)	

Table 2: demonstrates the peri operative co- morbidities among the patients according to surgical technique

Overall, postoperative rate of complications was also insignificantly different in all groups(Group I: 13.3 %, Group II:17.5 %, Group III: 18 % with *p* = 0.332).

The postoperative complications encountered included superficial or deep wound infection in two cases from Group I, two patients from Group II and three patients from Group III (one of them died due to septic shock). Partial foot drop was noticed in two patients in group III. Deep venous thrombosis and pulmonary embolism occurred in four patients from Group II and III (one of patient died and the other was treated); while sciatic pain continued to be problematic in two cases from Group I, three from Group II and two from Group III.

In this group study only two cases deceased; one in group II died of severe pulmonary embolism while the second one died of severe sepsis in group III. With total mortality rate at 4 % in all groups.

However, the post-operative complications had improved dramatically over the time except in the patient with complete foot drop in group III.

**Fig. 1:**
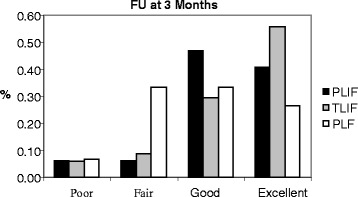
The demographic distribution of all cases in each group based on the provisional criteria at different follow-up times. After three months

**Fig. 2:**
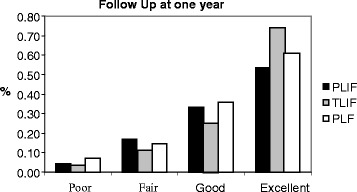
The demographic distribution of all cases in each group based on the provisional criteria at different follow-up times. After one year

**Fig. 3:**
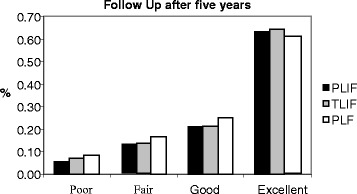
The demographic distribution of all cases in each group based on the provisional criteria at different follow-up times. After five years

**Fig. 4:**
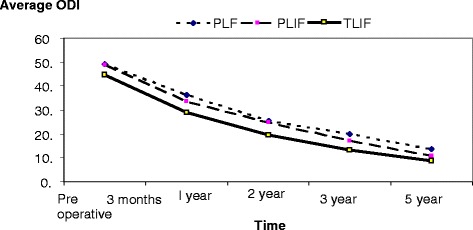
Illustrates the long term clinical improvement in ODI in the three groups

**Fig. 5:**
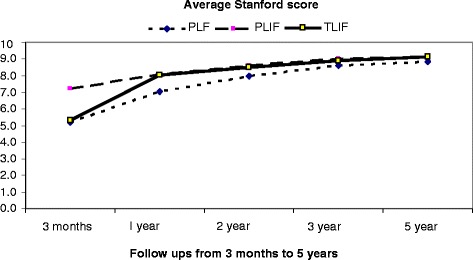
Shows a significant clinical improvement with periods of follow up according to Stanford scores

The demographic distribution of all cases in each group based on the provisional criteria at different follow-up times is shown in (Figs. 1, 2 and 3). While (Figs. 4 and 5) shows the change in ODI and Stanford scores over time in the three groups. The Pre-operatively ODI score was not significantly different among the three groups (*p* = 0.547) . While there was a significant decrease in the ODI score over time in the three groups with substantial p-value (p-value for trend < 0.005), with trivial difference in the change in the mean ODI among them at different follow-up times.

In terms of radiological evaluation that included plain and lateral dynamic radiographs to assess fusion. It was evident in 27 out of 30 in Groups I, 37 out of 40in group II and 47 out of 50 in group III with a rate of 90 %, 92.5 % and 94 %, respectively.

## Discussion

Variable surgical techniques with and without instrumental fixation are widely suggested to deal with different spinal pathologies. Many authors recommended anterior approaches others posterior. However, The posterior approaches have gained more popularity among spine surgeons with satisfactory results and fewer complications as compared with anterior approaches. Posterior lumber fusion (PLF), Posterior lumber interbody fusion (PLIF) and Transforminal lumber interbody fusion (TLIF) are frequently used to relieve pain and nerve compression in patients with degenerative disc disease, lumber canal stenosis spondylolisthesis, scoliosis failed back surgery and traumas [10, 23, 24].

Degenerative Lumber spine disc disease is a common cause of chronic and disabling pain that usually requires intervention. Several surgical techniques are recommended to overcome this problem. arthrodesis without instrumentation can be used, but this has not been proved to be an alternative option as rate of non-union is considerable on the long term follow up and in cases with osteoporosis or when multiple levels used. Furthermore, posterior lumbar interbody fusion (PLIF), transforaminal lumbar interbody fusion (TLIF), and posterolateral fusion and posterior instrumentation (PLF) are commonly suggested when PLF is combined with rigid stabilisation, it gives better fusion although there is a chance of graft resorption [17, 23, 24]. Posterolateral and interbody fusion technique is more rigid construct that can be achieved immediately after surgery, it also provides 360° fusion mass and protects the posterior instruments from straining PLIF has been widely used to treat degenerative spinal column diseases with canal stenosis [9–12].

The results of TLIF were first published in 1998 by Harms et al., he operated on 191 patients between 1993 and 1996 for variable spinal diseases including sponylolisthesis, post disc surgery, scoliosis and lumber canal stenosis with excellent results and low co morbidities [25]. However, TLIF is commonly effective in patients with chronic back pain with or without pain radiation and mild to moderate canal stenosis with satisfactory results in two thirds of cases. Better outcome may be significant with careful patient selection. The technique of interbody fusion application is biomechanically crucial, as it preserves the sagittal plane and provides the normal mechanical station of the whole spine, pelvis and lower limbs [24, 25].

Many studies reported the outcome of each technique individually; just a few studies have compared the results of these three surgical techniques. Audat et al. concluded that all techniques are similar and none of them is superior to the other. The best biological fusion was observed with TILF as evident with the radiological follow up. To achieve better outcome; he recommended to restrict the indications of each technique used to deal with degenerative spinal disease based on the patient’s symptoms and signs [26–29].

In this study, We observed a significant improvement on the long term follow up from ‘fair’ to ‘good’ and from ‘good’ to ‘excellent’ in all groups (Figs. 1–3). In Group III, less of the epidural space was dissected, and the lamina and facet joint were also preserved, which may account for the slightly higher number of ‘excellent’ outcomes and less late complications in Group III patients compared to the other two groups, but this was statistically insignificant. Although Group III showed the highest fusion rates among the three groups, as there was a wide interbody area, lamina and facet joint were preserved on one side and the intertransverse processes provided a wide area for fusion. Although TILF has shown better clinical on the short term follow up but we did not find any significant differences among the three treatment groups in the long term follow up in terms of clinical improvement, we concluded that all techniques can be effective with same results particularly when a certain technique implied for a certain case.

## Conclusions

This study shows no difference in intra- or postoperative complications among the three treatment groups, despite some fatalities observed in some groups and irrelevant to the technique. Besides that, there was no statistical difference in ODI, Stanford score and local clinical criteria among the three groups, although the radiological fusion rate was found better with TLIF (91.9 %). We recommend using any surgical technique to manage DDD with no privilege of any technique to the other, better outcome can be achieved with certain patient selection and the experience of the surgeon.
